# Acknowledgement to Reviewers of *Gels* in 2017

**DOI:** 10.3390/gels4010006

**Published:** 2018-01-10

**Authors:** Gels Editorial Office

**Affiliations:** MDPI AG, St. Alban-Anlage 66, 4052 Basel, Switzerland

Peer review is an essential part in the publication process, ensuring that *Gels* maintains high quality standards for its published papers. In 2017, a total of 49 papers were published in the journal. Thanks to the cooperation of our reviewers, the median time to first decision was 21 days and the median time to publication was 47 days. The editors would like to express their sincere gratitude to the following reviewers for their time and dedication in 2017:

Abdellatif, AbdelmohsanLeporatti, StefanoArgia, KatsuhikoLiefeith, KlausBandyopadhyay, SulalitMajor, IanBednarz, SzczepanMathew, Aji P.Ben Messaoud, GhaziMedronho, BrunoBernards, MatthewMerino, SoniaBetancourt, TaniaMiravet, Juan F.Bokias, GeorgiosMontenegro, LuciaBong, Ki WanMoth-Poulsen, KasperBouropoulos, NikolaosMoura, Maria J.Cerroni, BarbaraMuench, FalkChandrasekaran, SwethaNair, DevathaChiessi, EsterNgai, ToChronopoulou, LauraNonappa, NonappaCui, HaitaoOddo, LetiziaCustodio, CatarinaOhsedo, YutakaCzajkowska, AnetaOkesola, BabatundeDelbecq, FredericPaduano, LuigiDíaz Díaz, DavidPalocci, CleofeDomenici, FabioPapadakis, ChristineDouglas, Jack F.Paradossi, GaioDräger, GeraldPatterson, JenniferDupin, DamienPereira, LuísEceiza, ArantxaPouponneau, PierreErk, KendraPuiggali, JordiEsquivias Fedriani, LuisRatke, LorenzFranke, DanielaRaucci, Maria GraziaFu, QiuyunRibeiro, ClarisseGarcia-Gonzalez, Carlos A.Rovigatti, LorenzoGentile, PiergiorgioRubio-Hernández, Francisco J.Ghahremaninezhad, AliRusso, LauraGhugare, Shivkumar V.Salipante, PaulGohy, Jean-FrancoisSavo, Domenico FabioGormley, AdamSchiraldi, David A.Gradzielki, MichaelSerban, MonicaGuerra, GaetanoSher, PraveenHellweg, ThomasSlosarczyk, AgnieszkaHeras, AngelesSmirnova, IrinaHuang, WeiminSong, ChangsikIatridi, ZacharoulaSteed, JonathanIatrou, HermisStokke, Bjørn T.Ijima, HiroyukiSun, YuanIkeda, MasatoTananaiko, OksanaIno, KosukeTaylor, JoanJain, GauravTeramoto, YoshikuniJohn, Vijay T.Thordarson, PallKadlubowski, SlawomirToca-Herrera, José LuisKali, GergelyTomasini, ClaudiaKalyon, Dilhan M.Torres, María D.Kato, TadashiTunesi, MartaKim, JongseongUrayama, KenjiKrebs, MelissaVolker, KörstgensKuang, HuihuiWang, Hui-LiangKuckling, DirkWeiss, Richard G.Kundu, SantanuWinkler, Roland G.Kyle, StuartYamanaka, MasamichiLapitsky, YakovYang, YonggangLarraneta Landa, EnekoYoon, JinhwanLázár, IstvánYu, JiashingLee, SeungaeYu, XinjunLenfant, GillesZaccone, Alessio

